# 2297. Trends in Respiratory Pathogens Detected by Using a Multiplex PCR Panel during the Different COVID-19 Pandemic Phases in Taiwan

**DOI:** 10.1093/ofid/ofad500.1919

**Published:** 2023-11-27

**Authors:** Yu-Lung Hsu, Yan-Yi Low, Chiung-Tzu Hsiao, Yu-Chang Chang, Hong-Mo Shih, Hsiao-Chuan Lin, Kao-Pin Hwang, Po-Ren Hsueh

**Affiliations:** Division of Pediatric Infectious Diseases, China Medical University Children’s Hospital, Taichung, Taiwan, Taichung, Taichung, Taiwan; China Medical University Children’s Hospital, China Medical University, Taichung, Taiwan, Taichung, Taichung, Taiwan; Department of Laboratory Medicine, China Medical University Hospital, Taichung, Taiwan, Taichung City, Taichung, Taiwan; Department of Laboratory Medicine, China Medical University Hospital, Taichung, Taiwan, Taichung City, Taichung, Taiwan; School of Medicine, College of Medicine, China Medical University, Taiwan; Department of Emergency Medicine, China Medical University Hospital, Taichung, Taiwan; Department of Public Health, China Medical University, Taichung, Taiwan, Taichung City, Taichung, Taiwan; Division of Pediatric Infectious Diseases, China Medical University Children’s Hospital, Taichung, Taiwan, Taichung, Taichung, Taiwan; Division of Pediatric Infectious Diseases, China Medical University Children's Hospital, China Medical University, Taichung, Taiwan; School of Medicine, China Medical University, Taichung, Taiwan, Taichung City, Taichung, Taiwan; Departments of Laboratory Medicine and Internal Medicine, China Medical University Hospital, Taichung, Taiwan; School of Medicine, China Medical University, Taichung, Taiwan; PhD Program for Aging, School of Medicine, China Medical University, Taichung, Taiwan, Taichung City, Taichung, Taiwan

## Abstract

**Background:**

The COVID-19 pandemic has greatly affected other respiratory infectious diseases. Taiwan's government implemented strict control measures initially, followed by relaxing measures, which may have impacted the incidence and distribution of other respiratory pathogens. This study aims to investigate the epidemiology of respiratory pathogens in Taiwan during different COVID-19 pandemic phases.

**Methods:**

This retrospective study enrolled patients who visited China Medical University Hospital and China Medical University Children's Hospital in Taiwan between January 2020 and December 2022 during the COVID-19 pandemic. The respiratory tract specimens of these patients were detected using a commercial multiplex PCR-based panel assay that could identify 19 respiratory viruses and 4 bacterial pathogens. The study analyzed and compared the distribution patterns of respiratory pathogens among patients across three phases of the COVID-19 pandemic in Taiwan. These phases were divided into the transmission-blocking phase (period 1: January 2020 - December 2020), the transitional phase (period 2: January 2021 - December 2021), and the coexistence phase (period 3: January 2022 - December 2022).

**Results:**

A total of 5,820 patients were included in the study. Of which,1,780 (30.6%) were positive for at least one respiratory pathogen. The most commonly detected virus was human rhinovirus/enterovirus (939/2492, 37.7%), followed by RSV (406/2492,16.3%), parainfluenza 3 (402/2492,16.1%), and adenovirus (261/2492,10.5%). Co-detection of multiple respiratory pathogens was observed in 9.5% of patients. Human rhinovirus/enterovirus was mainly detected throughout the three periods, peaking in the summer and winter. Human metapneumovirus and adenovirus were predominant during period 2, peaking in the summer, while parainfluenza 3 and RSV were predominant during period 3, peaking in the winter.

**Figure 1.**
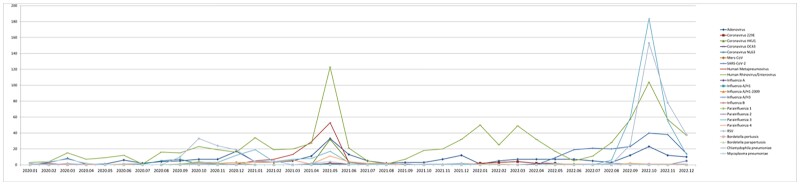
Monthly respiratory pathogen distribution during three years of COVID-19 pandemic

**Figure:**
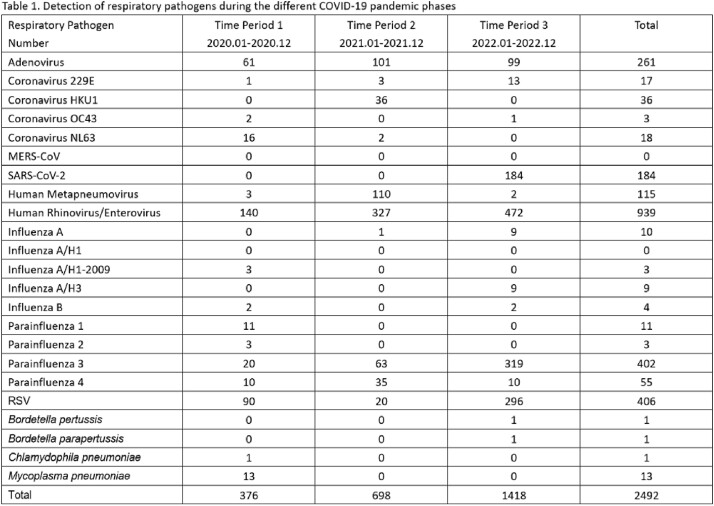

**Figure 2.**
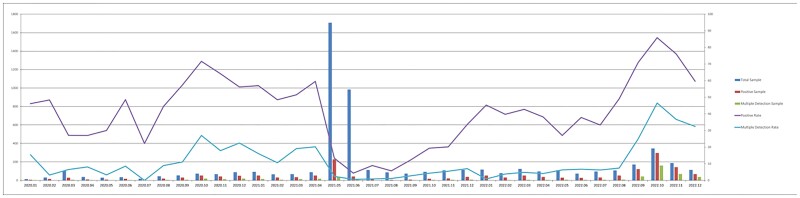
Monthly distribution of sample and detection rate during three years of COVID-19 pandemic

**Conclusion:**

This study showed significant changes in the detection rates of non-SARS-CoV-2 respiratory pathogens during different phases of the COVID-19 pandemic in Taiwan. These findings highlight the importance of continued surveillance of respiratory pathogens beyond COVID-19 to better understand their epidemiology and inform public health interventions.

**Disclosures:**

**All Authors**: No reported disclosures

